# Multi-Channel Multi-Radio Using 802.11 Based Media Access for Sink Nodes in Wireless Sensor Networks

**DOI:** 10.3390/s110504917

**Published:** 2011-05-04

**Authors:** Carlene E.-A. Campbell, Shafiullah Khan, Dhananjay Singh, Kok-Keong Loo

**Affiliations:** 1 School of Engineering and Information Sciences, Middlesex University, London, NW4 4BT, UK; E-Mail: carlnjam@gmail.com; 2 Kohat University of Science and Technology, Pakistan; E-Mail: skkust@hotmail.co.uk; 3 Division of Fusion and Convergence of Mathematical Sciences, National Institute for Mathematical Sciences (NIMS), Korea

**Keywords:** WSN, multi-channel, multi-radio, IEEE 802.11, nodes, channels

## Abstract

The next generation surveillance and multimedia systems will become increasingly deployed as wireless sensor networks in order to monitor parks, public places and for business usage. The convergence of data and telecommunication over IP-based networks has paved the way for wireless networks. Functions are becoming more intertwined by the compelling force of innovation and technology. For example, many closed-circuit TV premises surveillance systems now rely on transmitting their images and data over IP networks instead of standalone video circuits. These systems will increase their reliability in the future on wireless networks and on IEEE 802.11 networks. However, due to limited non-overlapping channels, delay, and congestion there will be problems at sink nodes. In this paper we provide necessary conditions to verify the feasibility of round robin technique in these networks at the sink nodes by using a technique to regulate multi-radio multichannel assignment. We demonstrate through simulations that dynamic channel assignment scheme using multi-radio, and multichannel configuration at a single sink node can perform close to optimal on the average while multiple sink node assignment also performs well. The methods proposed in this paper can be a valuable tool for network designers in planning network deployment and for optimizing different performance objectives.

## 1. Introduction

Wireless sensor networks are renowned for having limited transmission ranges and organizing themselves in an *ad hoc* fashion. When a wireless sensor cannot reach the receiver directly it relies on other sensor nodes to relay data between them. They are assumed to have constrained energy sources because they rely on batteries which may or may not be replaceable. Wireless sensor networks consist of large number of sensors [1–6], each equipped with the capability of sensing the physical environment, data processing and communicating wirelessly with other sensors. The number of nodes in a sensor network is significantly larger than other wireless networks; the difference can be of several orders of magnitude. Sensors are usually low-cost devices with severe constraints with respect to energy source, power, computation capabilities and memory. Sensors are usually densely deployed and the probability of failure is usually much higher. The sensors are usually stationary rather than constantly moving, however the network topology can still change frequently due to node failure.

In our previous work [7,8] we studied multichannel communication based on the 802.11 DCF over a single radio for wireless sensor networks in order to improve its communication performance on throughput, end-to-end delay and channel access delay. The proposed backoff algorithm, MC-DCF, allows node to have access to multiple non-overlapping channels by accessing channels dynamically through channel switching after a set threshold has been met. We focus on high data rate streaming that would be considered for a sensor surveillance system that would be deployed for organization, parks, and vehicular traffic, not for remote monitoring. For this reason we had considered static nodes that are always powered and as such the depletion of battery life is not considered. In our previous papers we analyzed MC-DCF performance of the non-overlapping channels on the mentioned metric against other protocols, we studied the impact of the number of non-over-lapping channel in the 2.4 frequency band of the 802.11 network, we analyzed the density of the network, we examined the effect of the sink node receiving data directly from sources within its range and finally a performance analysis of 802.11a/b/g was done. We had observed that MC-DCF performed poorly when receiving data at the sink node due to a single radio that had to be constantly switching channels and consequently more work needed to be done in this area to improve the performance at the sink. We also observed that at 50 m range with 10 Mbps all networks performed well. In this paper we have focused on improving the severe degradation that was experienced at the sink node. We look at the relationship between communication links from a graph based approach; this approach has been formally modeled by researchers and we considered the following to improve our model:

- Multiple sinks with single radio
- Single sink with multiple radios
- Single sink with multi-radios in a round robin fashion
- Multiple sink with multi-radios

These solutions improve contention, limited bandwidth and interference which are some of the barriers preventing successful delivery of large amount of data. The multichannel MAC protocol designed to provide high throughput and high delivery ratio during high rate traffic in the IEEE 802.11 network that normally use as Access Points (APs) or at cluster heads in sensor networks. The WSN in our studies uses constant bit rate (CBR) for streaming data that mimics surveillance and multimedia sensor network data that is foreseen to pose significant problems operating in smaller networks such as IEEE 802.15.4 and IEEE 802.11n when it becomes popular in the future. Exploring the best possible use is a challenging problem, but we foresee the future of WSN operating on hand held devices such as mobile phones to sense and interact the surrounding environment for safety of individuals travelling in areas such as parks and or lonesome areas that trigger alerts to security personnel.

A lot of works have been devoted to the problems of sensor networks but not for the high data rates encountered in 802.11 networks as in our work. These works looked at topology control [9,10], power management [11,12], energy awareness and optimal routing [13–20]. Recent focus has shown concentration in multichannel assignment [21–34]. Multichannel communication is an efficient method to eliminate interference and contention on wireless medium by enabling parallel transmissions over different frequency channels. Most work on multichannel focus on:

- Static approach where each interface is fixed permanently or for a long period of time on a channel.
- Dynamic approach, which allows interfaces to switch channel from time to time to exploit the maximum channel diversity.
- Hybrid approach, where a fix interface on a channel is used for package control and exchange. The other interfaces are used to switch among remaining channels for data transmission. Other hybrid approaches consist of two parts; one part handles MAC issues and the second part is a distributed assignment algorithm.

The rest of the paper is organized is sections discussing related work, system model and problem formulation, simulation results and discussion, and, finally conclusions and future work.

## 2. Related Work

The multichannel multi-radio approach in IEEE 802.11 based wireless networks has been widely studied by a number of researchers and can be categorized as centralized and distributed approaches. The centralized approach has been further categorized as:

- Flow based
- Graph based
- Partition based

A centralized flow based approach presented in [22,23,36,37] proposes a centralized joint channel assignment and multi-path routing algorithm. The channel assignment algorithm first considers high load edges. The routing algorithm uses both shortest path routing and randomized multi-path routing which is a set of paths used between any pair of communicating nodes. The joint channel assignment and multi-path routing algorithm proceeds in an iterative fashion. However, their algorithm is based on heuristics and the worst performance bound on its performance is not known. In addition to their scheme no guarantees on fair allocation of bandwidth is provided. However, a simulation study shows that by deploying just two NICs per node, it is possible to achieve an eight-fold improvement in the overall network goodput, when it is compared with the conventional single-NIC-per-node in wireless *ad hoc* networks. This is inherently limited to one single radio channel. In [22] they assume that there is no system or hardware support to allow a radio interface to switch channels on a per-packet basis. They also assume a radio interface is capable of switching channels rapidly and is supported by system software. Their evaluation demonstrates that our algorithm can effectively exploit the increased number of channels and radios, and performs much better than the theoretical worst case bounds. Kodialam *et al.* [36] define a standard multi-commodity flow problem on a MC-MR network; they assume that the traffic demand for different source destination pairs is given in the form of a rate vector. In their algorithms, it is not clear if it is possible to jointly optimize routing, link channel assignment and scheduling in a distributed manner.

A centralized, graph based approach has been proposed in [38–40], where links and nodes are considered as edges and vertices of a graph respectively in formulating radio and channel assignment by assigning edges to vertices. The limitation of these methods is that it is very difficult to capture network load information with a graph model. Network flow based centralized approaches can be found in [22,23] and [36], where multi radio multichannel is modeled based on network flows to overcomes the limitations associated with graph based approaches. These approaches are not realistic as constant traffic sources are assumed all the time while network traffic can be bursty in nature. Mahesh *et al.* [38] have considered the channel assignment, radio-channel mapping problem in multi-radio wireless mesh networks. They have argued that a traffic-independent channel assignment that provides a connected and low interference topology can serve as a basis for dynamic, efficient and flexible utilization of available channels and radios. In [28] a simple approach to address this issue is common channel assignment (CCA) which assumes that radio interfaces at each node are assigned to the same set of channels. This leads to inefficient channel utilization in the typical case where number of interfaces per node is fewer relative to the number of channels. Another graph based approach studied in [40] on an extensive evaluation via simulations shows that multi-radio scenarios, yields performance gains in excess of 40% compared to a static assignment of channels. In [41], the authors have addressed co-existence of heterogeneous interfaces and introduced a radio based novel graph model which captures the heterogeneity of interfaces.

A partition approach [42] designs a new algorithm that takes advantage of the inherent multi-radio capability of Wireless Mesh Networks (WMNs). They partition a network in a manner that not only expands the capacity regions of sub-networks, but also allows distributed algorithms to achieve the capacity regions. However, they will need to allow dynamic channel allocation that will require the channel allocation algorithms for online and distributed operation.

A distributed gateway centered multi-radio multichannel approach has been developed by [43] and [44] where mesh gateways are considered as sinks and sources of data. These approaches consider the coexistence of more than one radio interface of the same homogenous standard on a mesh router and use more than one available orthogonal channel. In [41], its authors have addressed co-existence of heterogeneous interfaces and introduced a radio based novel graph model which captures the heterogeneity of interfaces. They have also formulated scheduling, routing and channel assignment as an optimization problem. Their results show improvement in network capacity while preserving node level fairness. In [45] the given network consists of a set of stationary wireless routers where some of them also act as gateways to the Internet. They assume that the paths between the routers and the gateways have been pre-determined, for example, the neighbor-to-interface binding mechanism in [43] which can be used to determine the paths and the logical topology of the network. In their work the implementation can either be centralized or distributed. For distributed implementation, each node is responsible for assigning the optimal channels to some links. One of the distinct advantages of this algorithm is that it has the ability to assign the non-overlapping channels and also the partially overlapping channels. This allows the IEEE 802.11 frequency band to be fully utilized.

## 3. Problem Formulation

We study the problem of designing an efficient and distributed algorithm that overcomes the severe degradation at the sink node when using single radio to switch to multiple channels. We aim to achieve better performance in terms of delay, throughput and packet delivery ratio. We analyze the performance of our scheme via simulations, under realistic wireless scenarios. In our previous work [7,8] the single radio switches nodes to receive data from other sending nodes on different channels. The results obtained at the sink from the sending nodes have shown that MC-DCF performs very poorly. Source nodes close to the sink suffer from severe delay in delivering packets to the sink. This was as a result of more than one channel delivering packets to a sink node which operates on a single radio, where switching between channels has caused a build up of congestion in that a bottleneck has been created. We will address the problem at the sink node in the following ways.

## 3.1. Multiple Sink Nodes

We will increase the number of sink nodes to collect data from receiving nodes. The sink nodes will be equipped with a single radio and will be required to do channel switching in the same manner as in [7,8]. The advantage is that all data from senders will be received by more sink nodes located in strategic positions. This will eliminate the burden encountered by a single sink node.

**Figure 1.**
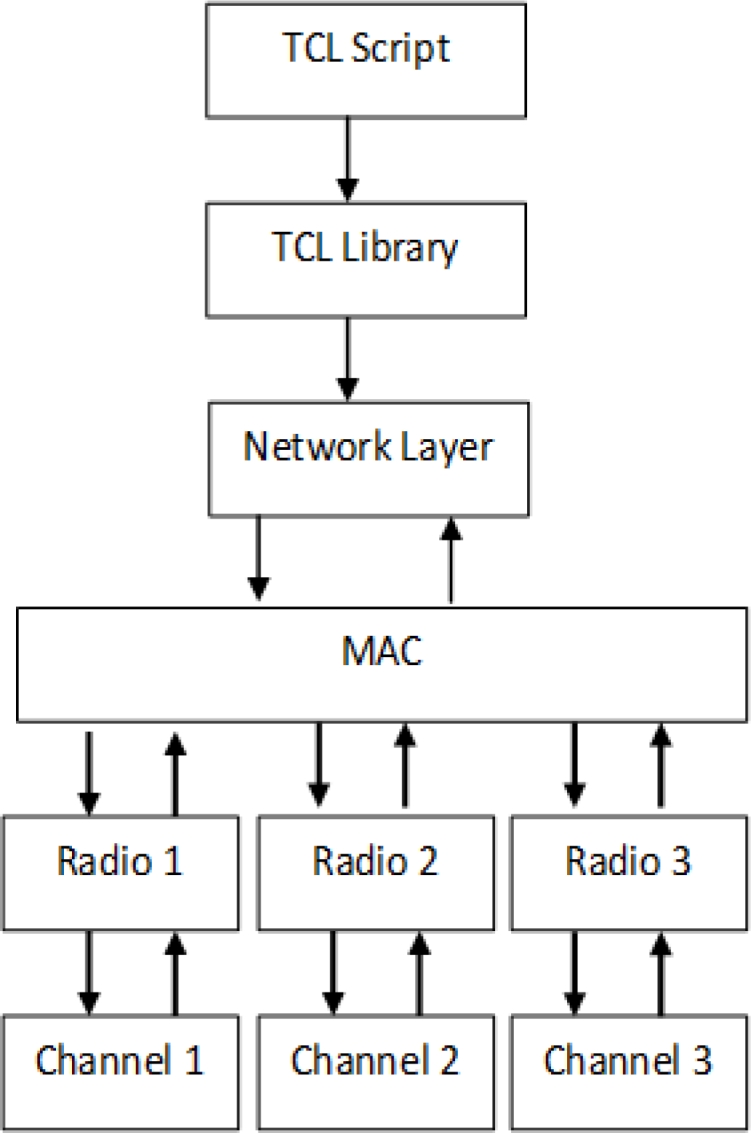
Design overview for multi-channel multi-radio.

We assign multiple radio interfaces in the sink node to receive data from each non-overlapping channel. The non-overlapping channels are a key technique to minimize signal interference and increase network capacity as such the multiple non-interfering channels are use to transmit data simultaneously and eliminates interference by another transmission. Each radio is assigned to a channel from each sending nodes. This will eliminate channel switching to all sending nodes by a single radio interface. Figure 1 shows modification at the MAC of the existing MACs protocol stack [46] this incorporate multiple radios, a new component is added to define the radio and radio number is set in the TCL script of the NS2. A new field is also created in the MAC into the packet header to index the channel object. This helps to achieve conflict free or reduce interference among neighboring nodes. To reduce communication interference nodes within communication range sense the network and conduct channel switching as illustrate in [7,8].

We take into consideration that it is not practical to have same number of radios and same number of channels at all times. The practicality of this depends on the network size. A medium to large sized network may have more nodes sending data to the sink.

By taking advantage of physical characteristics of the radio environment, the same channel can be reused by two or more nodes provided that the nodes are spaced sufficiently. To avoid co-channel interference we use non-overlapping channels. Since nodes are aware of all the channels at start-up and are able to switch channels based on a set of criteria from [7,8], the nodes sending packets to the sink are set to operate on a particular channel. All nodes are placed where they are in reach of the sink but separated by enough gaps between sending nodes. The reason for such arrangement is to ensure that radio interface switching between nodes on same channel will avoid co-channel interference.

Formally, channel assignments problems have been modeled as:

- Graph based [47,48], where the vertices *V* correspond to nodes and edges correspond to pairs of stations whose transmission areas intersect.
- Ring based [48] is considered as a form of vector where the size of the ring is a sequence of *n* vertices.
- Grid based [48] is considered a form of vector represent tessellations of a plane with regular polygon, where the size of the grid has row and column indexed from top to bottom and from left to right. The grid based can be classified as:
  ○ Bi-dimensional
  ○ Cellular
  ○ Honeycomb
- Tree based [48], where an undirected graph *T = (V, E)* is a free tree when it is connected and has exactly |*V*| – 1 edges.

These assignment techniques all use various vectors coloring problems which are based on arithmetic progressions to solve the channel assignment problems.

### 3.2. Multi-Radio Switching

We consider the WSN formed by static nodes and a sink node in [7,8] which are always powered to eliminate energy constraints, which are a major concern in WSN. The multichannel assignment will be presented in two ways. In the first way, each sink node is equipped with a single radio and can switch channels to receive data packets. In the other way, the sink node is equipped with multiple radio interfaces and has a distinct channel assign to each radio. However the nodes transmitting to the sink remain on the same channel and are not allowed to switch channels during transmission. In the case that changes or failure of any node or radio interface should occur, the sink node will update itself about these changes.

The multi-channel multi-radio (MCMR) problem can be modeled as an undirected graph where vertices denoting radios comprised the wireless network and a set of unidirected edges between vertices representing node link. The rationale is to prevent nodes on the same channel to attempt to send to the same radio interface. Nodes are numbered to prevent conflicts. Transmission take place interns base on number. Unidirected graph modeling [45] has been used to model channel assignment in wireless networks.

**Figure 2.**
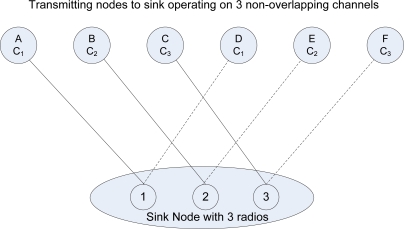
Sink Node with 3 Radios Receiving from 6 Transmitting Nodes on 3 non-overlapping channels (C1, C2, C3).

Consider a graph *G* = *(V, E)* where *V* is the set of wireless radios at the sink and L is the set of communication links between radios and transmitting nodes. For example, there are three radio interfaces at the sink node as illustrated in Figure 2, where *R_1...i_*, each radio interface corresponds to one or more edge nodes *Ni...x* but only one link can be active at any given time.

The broken line represents the inactive link and only becomes active when the associated radio switches to the active channel of that node. Each transmitting node is assigned to a channel and each radio only switches to a node on the same channel. A radio can receive data packets from more than one node on same channel. We derive the radio link as: *R_i_* ≤ *N_xn_*, where *x* and *n* is the node and channel number respectively. Consequently, only three links in Figure 2 can be active simultaneously, if *D*, *E*, and *F* attempt to transmit when *A*, *B*, and *C* is transmitting then a radio link conflict graph colouring problem occurs. To avoid a conflict graph colouring problem from occurring each Edge (*E*) that connected to a Vertex (*V*) is assigned a different colour. In the case where there are two *E* connected to each *V*, they are given colours True and False, this means that all the True can be transmitted at the same time and all the false become inactive. The color false becomes active when the radio link switches to the inactive edge.

G represents a graph, while *V* represents Vertex and *E* represents Edge(s):

- For this algorithm, *V* is a single Vertex while *E* can be 2 or greater (*E* ≥ 2).
- The equation *G* = (*V*, *E*) can therefore be replaced by *G* = (*V*, *E_I_*), where the subscript ‘*I*’ represents the variable for the number of Edges available.

The graph methodology is use to express the relationship between two communication links (represented by *E* in the equation) sending data to a single radio receiver (represented by *V* in the equation) non-simultaneously. Therefore at no time should both communication links be active to the common receiver/radio interface. The Algorithm 1 represents a system using two communication links or edges and its notation represented in Table 1. The objective is to ensure that only one communication link is active at any one time. The algorithm is laid out in a semi programming format.

We now consider the unidirectional links between the sink node and the transmitting nodes. Each source node is equipped with a single radio, but has access to multiple channels. The sink node which represents the server is equipped with a set of receiving radio interfaces. The capability for successful transmission between sender and receiver within the wireless range is denoted by a set of logical link (*L*) with *C* channels available. We define a binary vector of *L_l_* where *l* the number of links to a channel *C_n_* where *n* the number of channels as follows:

(1) $$L_{\left(l-1\right)}\;C\;+\;n\;=\;\{\begin{array}{c} 1,\;\text{if}\;l^{th}\;\text{link}\;\text{uses}\;\text{the}\;n^{th}\;\text{channel}\\ 0,\;\text{Otherwise} \end{array},\;\text{for}\;n\;=\;1\ldots ,\text{C};\;l\;=\;1\ldots ,\;\text{L}$$

Since only one channel can be assigned to each logical link *l*, between the lists of elements L_(l−1)_C + 1, L_(l−1)_C + 2 …, L_lC_ only one of them is equal to 1 and the rest are equal to 0. Therefore, we have the following equality constraints:

(2) $$\begin{array}{c} {\text{L}}_{\left(\text{l}-1\right)}\;\text{C}\;+\;1\;+\;\cdots \;+\;{\text{L}}_{\left(\text{l}-1\right)}\;\text{C}\;+\;\text{n}\;=\;1,\;\forall 1\;=\;1\;\ldots ,\;\text{L}\\ \Rightarrow \;AL\;=\;1 \end{array}$$

The dimension of the matrix *A* depends on the link on the same channel which uses the same radio interface. The active link is always equal to 1 and 0 otherwise. Therefore for each row in matrix *A,* one of the entries is equal to 1 and 0 otherwise.

The second constraint is imposed by the sink interfaces. The sink interfaces are the solution of the interface to the node binding problem. The constraint requires some links from a given node to use the same channel and radio. That is, if two links, *y* and *z* from a given node are assigned to use the same radio, then these two links need to be assigned to the same channel. This can be expressed as:

(3) $$\begin{array}{c} {\text{L}}_{\left(\text{y}-1\right)}\;\text{C}\;+\;\text{n}\;=\;{\text{L}}_{\left(\text{z}-1\right)}\;\text{C}\;+\;\text{n}\;,\;\forall \text{n}\;=\;1\;\ldots ,\;\text{C}\\ \Rightarrow \;BV\;=\;0 \end{array}$$

For each row in matrix *B*, two of the entries are equal to 1 and −1 respectively, and all other entries are equal to 0. The dimension of *B* depends on the number of linked pairs that share a common radio interface. The vector definition in (1) and the equality constraints in (2) and (3), together form the following non-empty feasible set:

(4) φ = {L : 1 ∈ {0,1} ∩ AL = 1 ∩ BL = 0}

where any of ϕ represent one feasible link assignment to a radio interface on same channel allocation.

Let’s consider any two arbitrary links d and e, and their associated elements in vector *V*. We define two *C* x 1 vectors as follows:

(5) $$\begin{array}{ccc} V_{d} & = & {\left[L_{\left(d-1\right)}\;C\;+\;1\;L_{\left(d-1\right)}\;C\;+\;2\;\ldots \;L_{d}\;C\right]}^{T}\\ V_{e} & = & {\left[L_{\left(e-1\right)}\;C\;+\;1\;L_{\left(e-1\right)}\;C\;+\;2\;\ldots \;L_{e}\;C\right]}^{T} \end{array}$$

We define *Ri* × *i* as the radio matrix at the sink. The element RAD ∈ [0,1] represents the radio interfa**c**e portion between nodes A and D to switch on the same channel *Cn*. R is a symmetric matrix and its diagonal elements all equal to 1. If node A and D are assigned to links d and e respectively we have:

(6) $$V_{d}^{T}\;R\;V_{e}\;=\;r_{AD}$$

For example, using the three non-overlapping channels *i.e.*, *C* = 3. *R* becomes a 3 × 3 unitary matrix. If two arbitrary links *d* and *e* are assigned the same channel, then $V_{d}^{T}\;R\;V_{e}\;=\;1$ otherwise, the product will equal to zero.

## 4. Simulation Works and Discussion

In our simulations we will be using the design model of our previous work [7,8], where we had modified the original 802.11 DCF to design an improved contention based MC-DCF protocol to perform channel switching in a multichannel single radio environment. We improve on this environment at the sink node, where previously we observed that channel switching among nodes by a single radio at the sink node causes severe degradation. This result in high packet delay and delivery ratio, we will be analyzing the performance at the sink of the MC-DCF protocol by simulations with NS2 [49]. This model uses a graphical user interface which provided various simulation scenarios. Users can select different network protocols, different topology and traffic model as well as users can design their own environment from the TCL script and the C++ library. This model includes radio parameter which incorporates interference information, traffic information and channel utilization information. The 802.11 radio model of the NS2 is used; this model has different topology and traffic generator. Different simulation scenarios will be studied according to three different performance metrics: aggregate throughput, delivery ratio and access delay. The sensor nodes are randomly placed in a 1,000 × 1,000 m^2^ area. The radio range is set to 50 m and simulations run for 500 s in each scenario. The radio bandwidth is 10 Mbps. We maintained these settings from our previous work [7–9] where we observed MC-DCF performed well in comparison to other range and rates. The number of nodes is 100. The numbers of channels used are three non-overlapping IEEE 802.11 ones that were used in our previous work [7,8] and which we use to compare with the current result and measure the performance improvement. Since the spectral mask only defines power output restrictions up to ±11 MHz from the centre frequency to be attenuated by 30 dB. It is often assumed that the energy of the channel extends no further than these limits. Our simulation uses static nodes to mimic surveillance sensor network with high data rate streaming that would be deployed for organization, parks, and vehicular traffic with nodes that are always powered, as such the energy consumption of the nodes are based on the power output ±11 MHz.

With the improvements made at the sink(s) to receive data directly from sending nodes within range we will be comparing these current simulation results obtained with the previous results from our work in [7,8] to determined the level of performances in percentages, that is new results minus the previous results divided by the previous results multiply by 100 (NR-PR/PR × 100). From the formulated solutions and equations derived to improve the degradation at the sink node encountered from our previous work we will consider the solutions that obtain better performance as the most feasible option to consider for our future MC-DCF.

### 4.1. Multiple Sinks with Single Radio

In our previous work [7,8], we examined the effect of the sink received data from sources within range that are sending data to be accepted. It was observed that the more sources sending to the single sink the more delays were encountered. In this scenario we have increased the number of sink nodes to receive data from sources within the ranges of the sink nodes. No modification to our MC-DCF protocol was made, except to increase the number of sinks to three with each having a single radio and doing channel switching as in our previous work. The simulation last for 500 seconds all nodes send CBR every 2 seconds.

**Figure 3.**
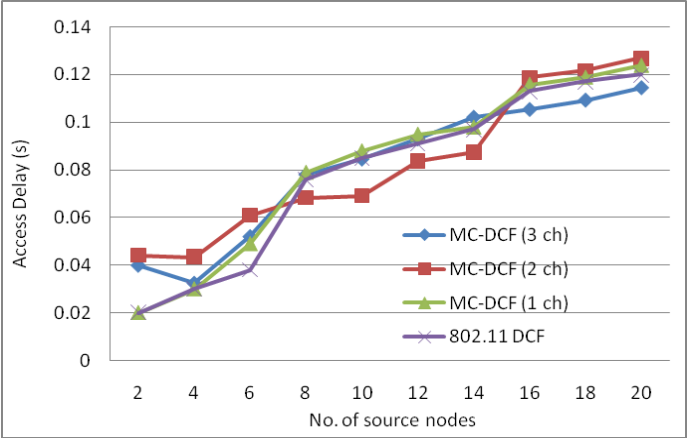
Delay impact from source nodes when using multiple sinks with single radio interface.

Figure 3 show the delay impact with the increase in sink nodes that are receiving data packets from the sending nodes within its range. We have observed that with three channels there has been a 53% reduction in delay at the sink side when compared to the high level of delay in our previous work [7,8] using only one sink node. In the same Figure 3 with two channels sending data from sources, there has been an approximately 32% delay improvement. Single channel and 802.11 DCF show little improvements. This indicates that single channel performance does not improve with increasing sink nodes as the decisions are based on the window size resetting, backing off, wait states and the fact that all nodes are contending for the same medium. MC-DCF with multiple channel switching and single radio interfaces can yield a better performance, when using multiple sinks compare to single channel which shows a better performance in previous work.

**Figure 4.**
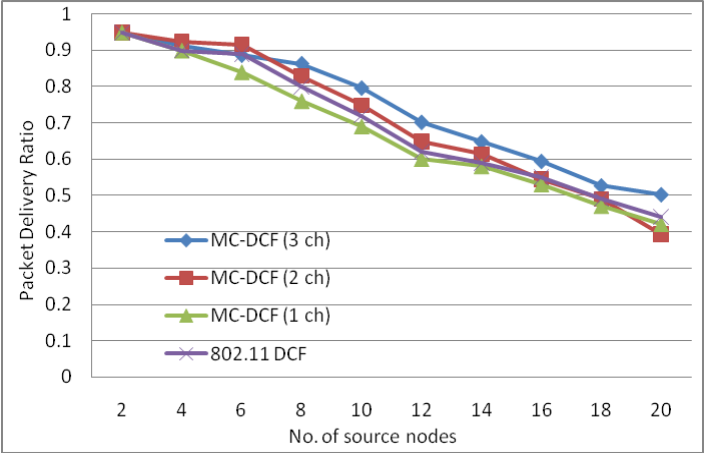
Delivery ratio impact from sources when using multiple sinks with single radio interface.

Figure 4 shows an improvement of over 41% for three channels with packet delivery ratio when the number of sink increases by three as compare to single sink node in our previous work. With two channels sending data from the sources to the sinks there has been improvement by over 25% comparing to the poor performance experienced with a single channel. Similarly, where the delay with a single channel shows no significant improvement, packet delivery ratio shows no major improvement.

**Figure 5.**
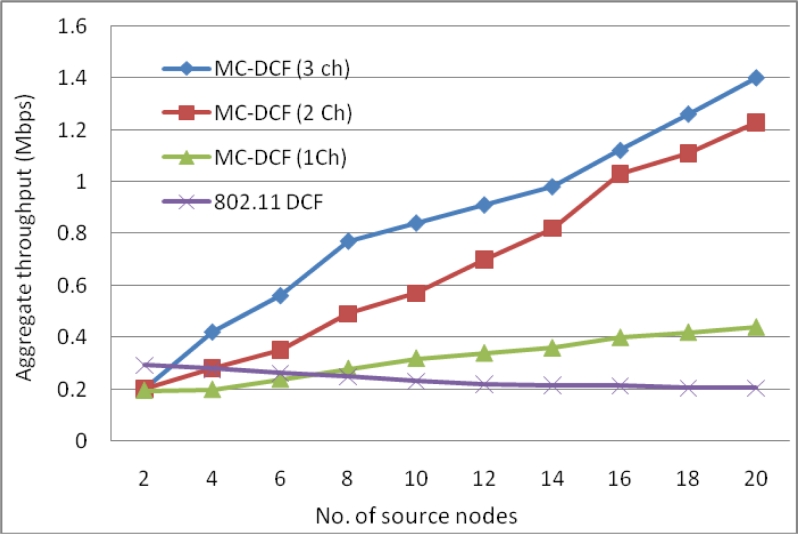
Throughput of the overall system using multiple sink nodes with single radio interface.

The aggregate throughput in Figure 5 of the overall system with source nodes sending to the sinks have shown that with three channels 38% more data have been delivered to the sink compared to that of a single channel. The single channel in all instances has not shown any significant improvement with increasing of sink nodes to receive data from the source nodes.

By analyzing the impact of MC-DCF with one to three channels in comparison with the original 802.11 DCF, we observed that increasing the number of sink nodes results in an improvement when two or three channels are used. There was little or no improvement using a single channel or the original 802.11 DCF which only operates on a single channel. The reason for this improvement is that each sink has less data to receive from the senders. The same amount of data simulated in previous work was going to a single sink node. The improvement proved that increasing the sink nodes gives a better performance as the traffic load has split to be received by more sink nodes. Therefore channel switching by a single radio has less data to retrieve, therefore less time is spent switching between channels from senders and the queuing of packet data has been reduced.

### 4.2. Single Sink with Multiple Radios

**Figure 6.**
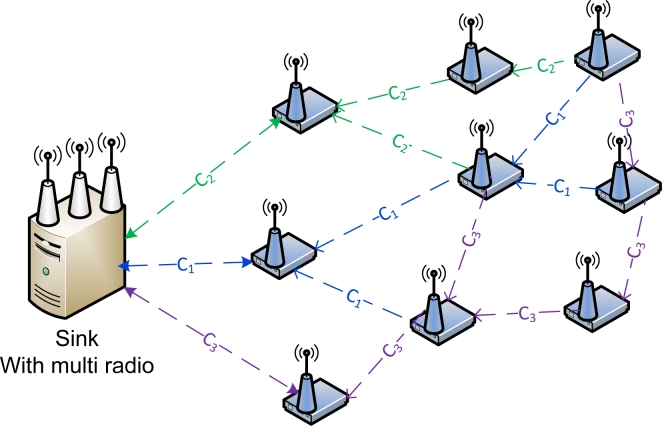
Single sink node with multiple radios.

In our second set of simulations, we use a single sink node and increased the number of sink radio interfaces to three. Figure 6 shows a sink node with three radio interfaces. We then assign each interface to a channel and assign three sending nodes on each channel to create a one to one mapping with the interface. In this case no channel switching is required. Each sender to the sink remains on said channel throughout the simulation. This allows constant flow between sending node and the radio interface.

**Figure 7.**
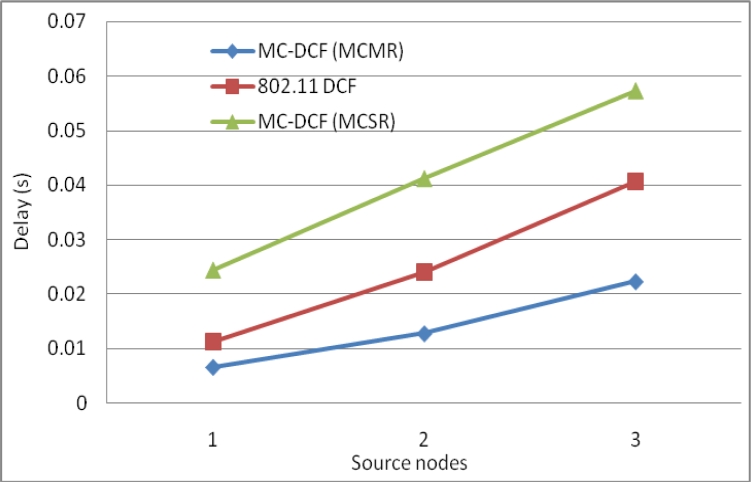
Delay impact with multichannel multi-radios communication at sink node.

Figure 7 shows the delay impact when MC-DCF uses a single sink with three radio interfaces to receive packet data which creates a one-to-one mapping in receiving data from sending nodes. MC-DCF with single radio from previous work had to perform channel switching to receive data when two or more non-overlapping channels are sending data to the sink. The result in our previous work showed that when two or more channels were used there was poor performance; the repeat of this performance is shown in Figure 7, except that only three sources were assigned to send data to the three interfaces, where each interface and each node is assign to one of the non-overlapping channels. However, when the one-to-one assignment is used we have seen over 40% success in improvement for delays. This outcome indicates that if we eliminate radio switching between channels and receive data flowing constantly from senders to the receiving radio interfaces we can improve the performance at the sink. However this would not be practical when network size increases, as we would need to constantly increase the radio interfaces at the sink and in addition, the limitation of non-overlapping channels would not make it feasible as there would not be enough non-overlapping channel to assign to radio interfaces.

**Figure 8.**
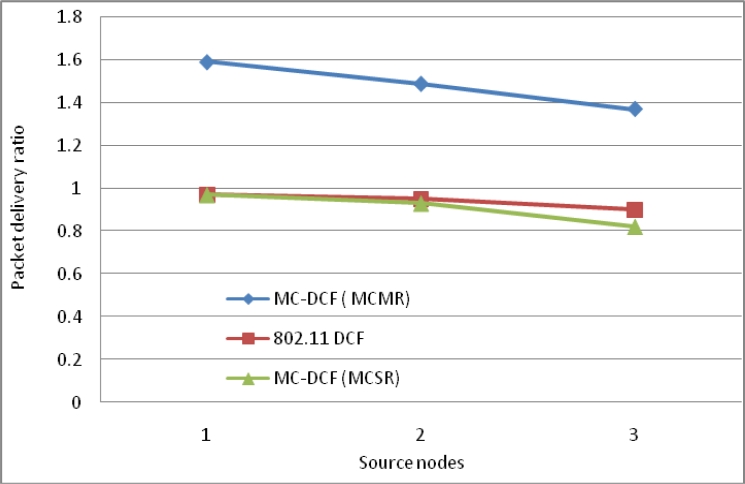
Delivery ratio impact with multichannel multi-radios communication at sink node.

**Figure 9.**
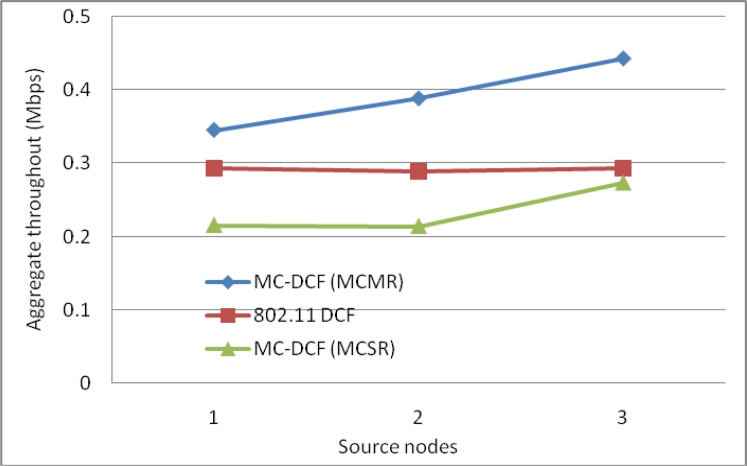
Throughput impact with multichannel multi-radios communication at sink node.

The packet delivery ratio in Figure 8 shows a similar improvement of approximately 46% for MC-DCF operating with multi-radios when compared to MC-DCF operating with a single radio as in our previous work. Each interface on a sending node is assigned to different non-overlapping channels. 802.11 DCF showed little or no improvement as this protocol was only designed to operate with a single channel. As mentioned before, the one-to-one assignments situation is not ideal for a large network as it would not be practical to have each radio interface assign to a non-overlapping channel from a sending node. Figure 9 also showed a 53% improvement in the one-to-one assignment with three non-overlapping channels for aggregate throughput. However for small parks and building areas this kind of implementation can be considered.

The one to one scenario demonstrated above is not practical in all instances, but will depend on the size of the network and the number of sending nodes directly to the sink. Ideally there will be more nodes sending to the sink that will create a one-to-many assignment, where many nodes are sending to the same radio interface. Sending nodes can be odd or even in numbers. We derived some equations to solve these scenarios for our next simulation. These equations are used when there are even transmitter (senders) to receiver (sink) and uneven transmitter to receiver ratios.

### 4.3. Single Sink with Multi-Radios in a Round Robin Fashion

In our third scenario we simulate multiple radios, multiple channels with even number of multiple sending nodes; however we define the following equations for the simulation. The equations have the capability to simulate odd or even sending nodes to the sink node. Sending nodes in these equations are referred to as transmitter and the radio interface as receiver.

Even Transmitter to Receiver ratio: (TX: RX, TX = Even Positive Integer and RX ≥ 2, TX ≥ 4).

Table 2 equations are containing only logical states (Active (1)/non-active (0)) values.

When there are even senders to the sink node, each radio interface shares an even number of sending nodes and the radio interface remain on the assigned channel with all nodes getting an even turn in transmitting its data to the sink in a round robin fashion which is explained later in this section.

Uneven Transmitter to Receive ratio: (TX: RX, TX = Uneven Positive Integer and RX ≥ 2, TX ≥ 5).

The equations in Table 3 demonstrate when there are uneven numbers of sending nodes to the radio interfaces at the sink node. When there are uneven numbers of senders only one sender can transmit at a given time; a logical state is considered where the active node sending is equal to one and all other senders are set to zero. In the equation we define seven senders and the odd sender is assigned in a sequential order where it receives an equal opportunity to send in respect of which channel it is assigned; however the uneven node has the option to switch channels but not during its period of transmission.

The equations contain only logical states (active (1) and inactive (0)) values. When a node is in its active state it is equal to one and when it is in a negative state it is equal to zero.

**Figure 10.**
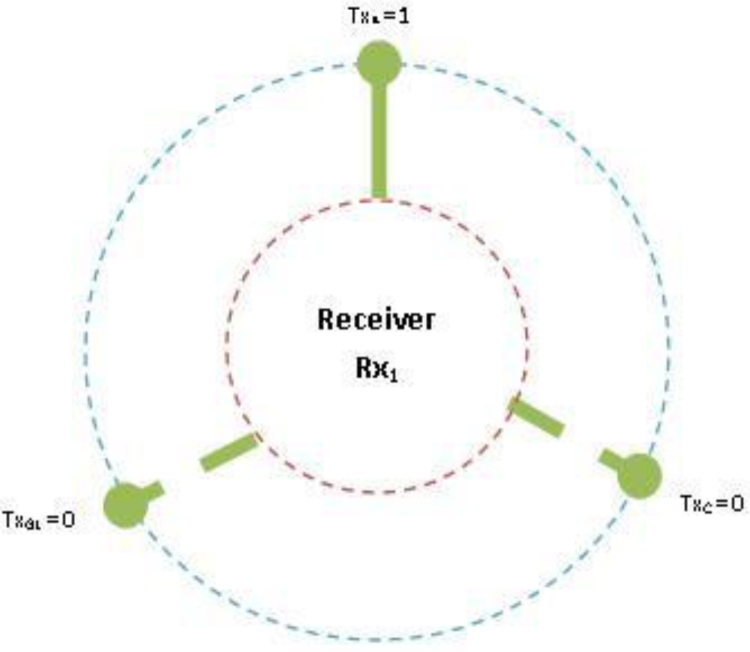
Radio receiving from node TXA.

- If a variable is negative then assign a logical 0 to the variable. For example –TXA = 0.
- If a variable is positive assign a logical 1. For example TXA = 1, therefore when RX1 = –TXA – TXC – TXG1, only TXA can be sending data to the receiving radio. Figure 10 illustrate a radio interface at the sink (receiver, RX1) accepting data from a node (transmitter, TXA = 1) represented by an unbroken link. Other nodes (TXC, TXG1) being zero are represented by broken links to illustrate their negative or inactive state.

Uneven Transmitter to Receive ratio: (TX: RX, TX = Uneven Positive Integer and RX >= 2, TX >= 5)

The equations contain only logical states; active (1) and inactive (0) values.

Table 4 equations yield the same outcome as Table 3 but have been expressed differently.

- We begin with senders to Receiver in a ratio of 6:3 which represents two transmitters to each receiver (2:1).
- By multiplying the output of ODD transmitter by the quantity of receivers we have created an even system where G is the sequential sending nodes that can be on any channel, the radio interface at the sink will sense G and update the number of receivers for data acceptance. However, G is not switchable to another channel when data is being sent to the receiver in a cycle.

**Figure 11.**
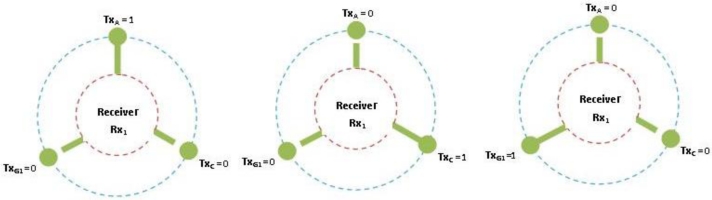
Round robin cycle.

These equations allow a round robin fashion; each radio operates as a single-Eulerian cycle, which listens to every node on same channel once in a cycle. When the radio is less than the number of sending nodes, we derive the logics so that radio operates in a round robin fashion. The round robin technique does not limit the number of radio interfaces, each interface will operate in the same way which will allow the sink node (s) to receive data from senders in a more effective and efficient manner. Take for example 6 sending nodes as illustrate in Figure 2 and Table 2 with even transmitter to receiver equations, assign to three non-overlapping channels, each radio interface will switch between two nodes per cycle. When the radio interface on channel 1 senses the first sending node, it will receive its data packets and then sense the medium for the next node on the same channel. It will switch to that the sending node receives its data and continues in that fashion throughout the simulation period. Figure 11 illustrates the round robin fashion where the radio interface(s) at the sink can receive from only one sender at any given time. When the interface is receiving the transmitter is equal to one which is represented by an unbroken link in the diagram and zero otherwise which is represented by the broken link.

In our third simulation scenario, we use a single sink with three radio interfaces; each radio interface is assigned to one of the three non-overlapping channels and six sending nodes to the sink, using the equations above in a round robin fashion. This assignment is semi-dynamic where two transmitting nodes are assign to the same channel and each radio interface at the sink switches among sending nodes on the same channel which gives a 2:1 ratio; two nodes transmit to one radio interface.

**Figure 12.**
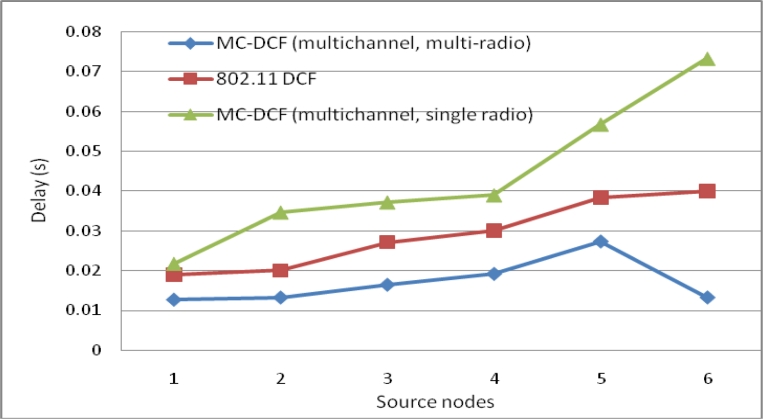
Delay impact comparison with one-many communications at sink node.

Figure 12 shows the delay impact among the six sending nodes and the radio interfaces at the sink. MC-DCF with the multichannel multi-radio (MCMR) assignment performs significantly better than MC-DCF with multichannel single radio (MCSR). When comparing the outcome with the performance of MCSR in our previous work, we have seen an improvement of over 55% for delay. This outcome indicates that with multiple radio interfaces MC-DCF can reduce the high delay encountered with a single channel as the senders need not queue to wait for access to a single radio interface. Instead senders can be distributed among several interfaces. This also reduces the extensive work of a single interface switching between several sending nodes to receive their data packages.

**Figure 13.**
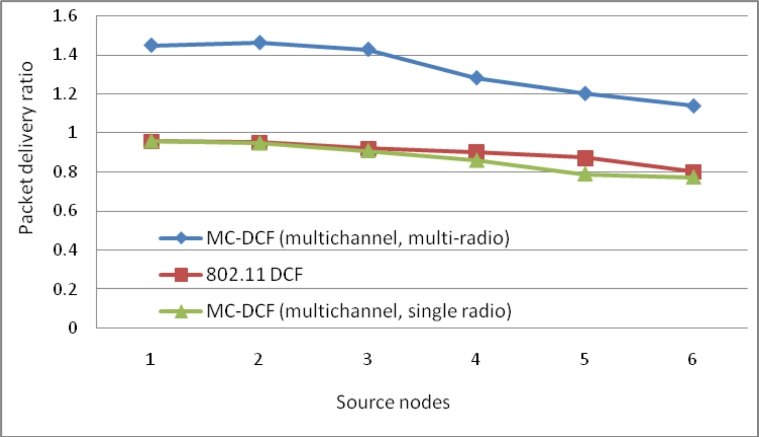
Delivery impact comparison with one-many communications at sink node.

**Figure 14.**
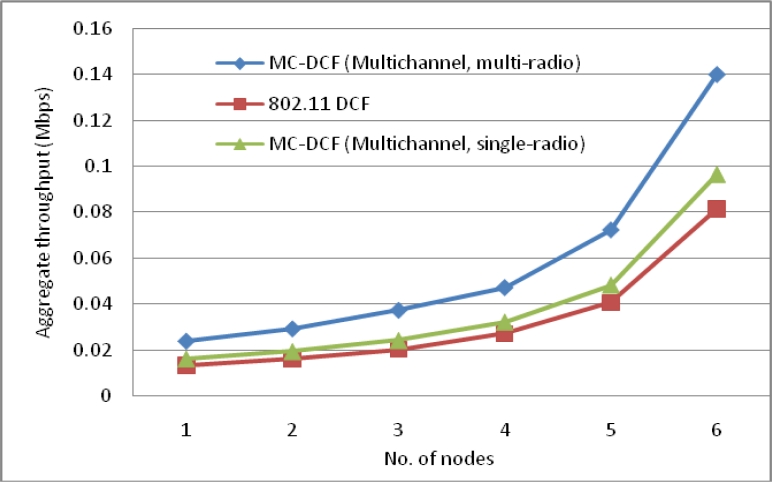
Throughput impact comparison with one-many communications at sink node.

In Figure 13, we observe a similar trend to that of delay where MCMR obtaining higher packet delivery ratio of over 51% compare to MCSR that perform very poorly from our previous work. Therefore, having multiple radio interfaces at the sink node to receive data packets from the three non-overlapping channels have improve the performance packet delivery and reduce the traffic load experience by a single radio interface. Figure 14, shows the overall aggregate throughput for the total amount of data delivered to the sink. MCMR show an overall better performance of 49.6% in comparison to that of MCSR offered load.

The single sink with multiple channels and radio interfaces scenario demonstrated above for MC-DCF has shown an improvement in performance over a single sink with multiple channels and single radio interface. There has not been any significant improvement in the 802.11 DCF, as it is a contention based protocol design to operate on a single medium, where all nodes contend for the single medium.

### 4.4. Multiple Sink Multi-Radios

In our previous scenarios we have simulated and analyzed the impact with:

- Multiple sinks, each with single radio
- Single sink with multi radio
- Single sink with multi-radio in a round robin fashion

Each scenario show some level improvement for MC-DCF when the sink node(s) obtain data from source nodes, compared to our previous work where the sink encountered severe degradation when receiving from sources by a single sink with single radio interface that has to constantly switching between sending node interfaces.

**Figure 15.**
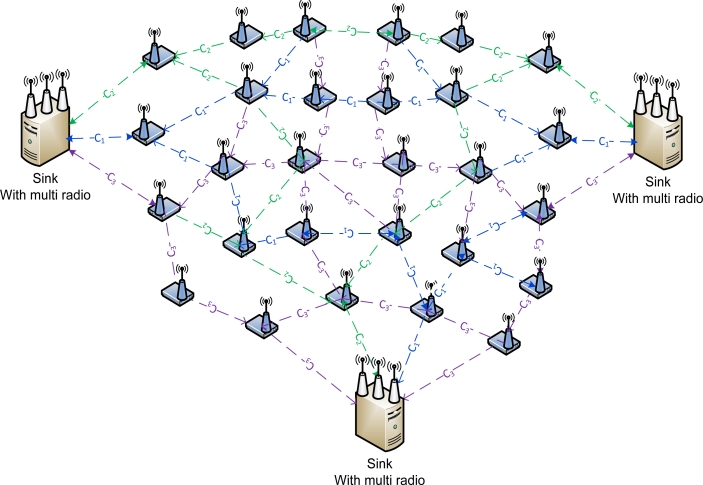
Multiple sink nodes with multiple radios.

In this scenario we will be analyzing the impact of data sending from sources to three sink nodes. Each sink will be equipped with three radio interfaces using the three non-overlapping channels in IEEE 802.11. Figure 15 shows a sensor network with multiple sink nodes each having three radio interfaces.

**Figure 16.**
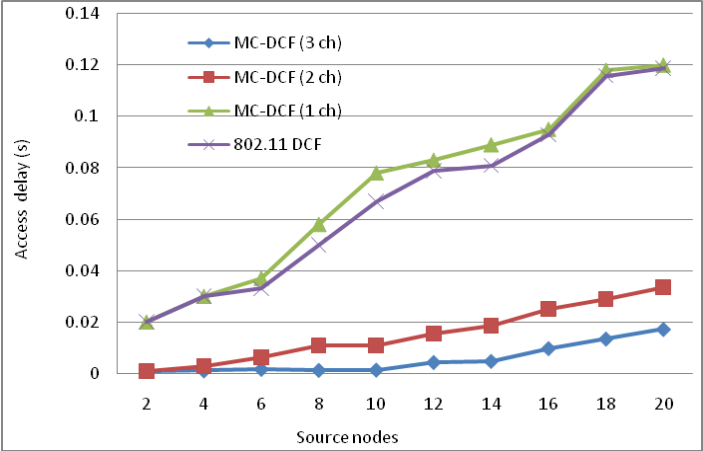
Delay impact from source nodes when using multiple sinks with multiple radio interfaces.

Figure 16 show delay impact with the increase in sink nodes and radio interfaces. We have observed that with three channels there has been a 96% reduction in delay at the sink side comparing to our previous work where the source node transmitting directly to the sink experience high level due to channel switching by the single radio interface. With two channels sending data from sources, there has been an approximately 87.4% delay improvement. Single channel and 802.11 DCF show little improvements. As mention previously single channel performance does not improve with increasing sink nodes or radio interfaces as the decisions are based on the window size resetting, backing off, wait states and the fact that all nodes are contending for the same medium. MC-DCF with multiple channel switching and multiple radio interfaces have yield a better performance when using multiple sinks in contrast to single channel and 802.11 DCF which shows a better performance in previous work.

**Figure 17.**
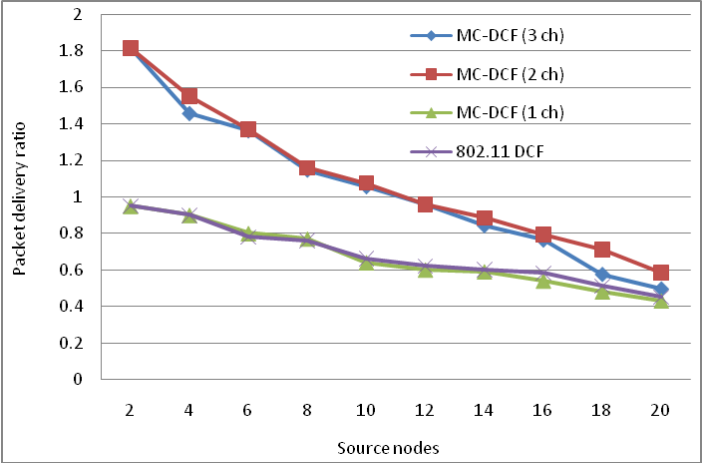
Delivery ratio impact from sources when using multiple sinks with multiple radio interfaces.

Figure 17 shows an improvement of over 90% for three channels with packet delivery ratio when the number of sink nodes and radio interfaces increase by three as compare to single sink node with single radio interface in our previous work. With two channels sending data from the sources to the sinks there has been improvement by over 81% compared to the poor performance experience with a single channel. Similarly, where the delay with a single channel shows no significant improvement, packet delivery ratio using single channel shows no major improvement.

**Figure 18.**
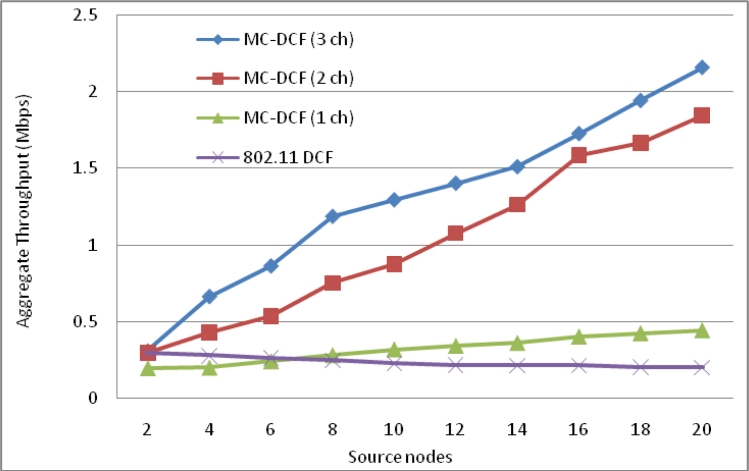
Throughput of overall system using multiple sink nodes with multiple radio interface.

The aggregate throughput in Figure 18 of the overall system with source nodes sending to the sinks have shown that with multiple sink nodes, channels and radio interfaces 92% more data have been delivered to the sink compare to that of single sink with single radio and single channel. Single channel and 802.11 DCF in all instances has not shown any significant improvement with increasing of sink nodes receiving data from the source nodes.

## 5. Conclusions

In this paper, we have addressed the poor performance encountered by the sink in our previous work. Our aim is to have WSN perform at an optimum rate in a multichannel environment of the 802.11 network for high data rates. In the future we foresee problems with 802.15.4 when the IEEE 802.11n standard becomes popular. We consider a WSN formed by static nodes with increasing the sink nodes and assigning multiple radio interfaces at the sink. We formulated solutions for solving multichannel multi-radio assignments at the sink by using graph techniques and a binary vector. We also created a number of equations to solve the scenarios of odd or even numbers of transmitting nodes sending data directly to the sink.

From the outcome of our simulations we prove that if we increase the number of sink nodes and/or increase the number of radio interfaces in the sink we can obtain a better performance at the sink, which results in an overall performance improvement within the network. The multi-radio interfaces assignment in the sink node will be the network to consider for the future, even though when we increase the sink nodes with single interface we have seen improvement in performance. The simulation scenario with three sink nodes, each equipped with three radio interfaces using the three non-overlapping channels in IEEE 802.11 is the network to be considered for future static WSNs with streaming data. We have seen from the simulation results that an average of over 90% improvement in performance can be achieved. As such, we can consider this kind of assignment to be more cost effective and energy efficient in the future.

In the future, we can consider setting up test bed to compare our empirical results with the test bed results. After further testing in the future areas that can benefit from the use of this application or relate to professional and industrial practice of this kind are transportation and traffic management, parks, built-up environment, festivals and sporting events such as Olympics and World Cup football, narcotics and border controls. We will also examine MC-DCF as a more cost effective solution when 802.15.4 is unable to operate in the 2.4 GHz with other standard such as 802.11n.

## Figures and Tables

**Table 1.** Notation Representation.

**Notation**	**Description**

N	Represent Nodes
x	The number of sending nodes to sink
C	Represent Channels
n	The number of non-overlapping channels
R	Represent Radio
i	The number of radios
L	Represent Links
*l*	The number of links
G	The graph
V	The vertex of the graph
E	The edge of the graph
I	The number available edges

**Table 2.** Equation for even sender to multiple radios at sink node on 3 non-overlapping channels.

**Senders (TX = 6)**	**Receivers (RX = 3)**	**Equation**

A = (TXA)	1 = (RX1)	RX1 = TXA – TXD: where TXD = 0
B = (TXB)	2 = (RX2)	RX2 = TXB – TXE: where TXE = 0
C = (TXC)	3 = (RX3)	RX3 = TXC – TXF: where TXF = 0
D = (TXD)	1 = (RX1)	RX1 = TXD – TXA: where TXA = 0
E = (TXE)	2 = (RX2)	RX2 = TXE – TXB: where TXB = 0
F = (TXF)	3 = (RX3)	RX3 = TXF – TXC: where TXC = 0

**Table 3.** Equation for uneven sender to multiple radios at sink node on 3 non-overlapping channels.

**Senders (TX = 7)**	**Receivers (RX = 3)**	**Equation**

A = (TXA)	1 = (RX1)	RX1 = TXA – TXC – TXG1: where TXC = 0, TXG1 = 0
B = (TXB)	2 = (RX2)	RX2 = TXB – TXE – TXG2: where TXE = 0, TXG2 = 0
C = (TXC)	1 = (RX1)	RX1 = TXC – TXA – TXG1: where TXA = 0, TXG1 = 0
D = (TXD)	3 = (RX3)	RX3 = TXD – TXF – TXG3: where TXF = 0, TXG3 = 0
E = (TXE)	2 = (RX2)	RX2 = TXE – TXB – TXG2: where TXB = 0, TXG2 = 0
F = (TXF)	3 = (RX3)	RX3 = TXF – TXD – TXG3: where TXD = 0, TXG3 = 0
G = (TXG)	*Sequential input to receivers{1 + 2 + 3}	RX1 = TXG1 – TXA – TXC: where TXA = 0, TXC = 0
		RX2 = TXG2 – TXB – TXE: where TXB = 0, TXE = 0
		RX3 = TXG3 – TXD – TXF: where TXD = 0, TXF = 0
		N.B. TXG1, TXG2, and TXG3 are switchable communication link from sender G going to each of the receivers. Therefore sender G has the same number of switchable time period to receivers. When G switch to a channel, G waits its turn to transmit then has the option to switch to another channel.

**Table 4.** Equation for uneven sender to multiple radios at sink node on three non-overlapping channels.

**Senders (TX = 7)**	**Receivers (RX = 3)**	**Equation**

A = (TXA)	1 = (RX1)	RX1 = TXA – TXC – TXG1: where TXC = 0, TXG1 = 0
C = (TXC)	1 = (RX1)	RX1 = TXC – TXA – TXG1: where TXA = 0, TXG1 = 0
G = (TXG)	* Sequential time period to receivers {1 + 2 + 3}	RX1 = TXG1 – TXA – TXC: where TXA = 0, TXC = 0
B = (TXB)	2 = (RX2)	RX2 = TXB – TXE – TXG2: where TXE = 0, TXG2 = 0
E = (TXE)	2 = (RX2)	RX2 = TXE – TXB – TXG2: where TXB = 0, TXG2 = 0
G = (TXG)	* Sequential time period to receivers {1 + 2 + 3}	RX2 = TXG2 – TXB – TXE: where TXB = 0, TXE = 0
D = (TXD)	3 = (RX3)	RX3 = TXD – TXF – TXG3: where TXF = 0, TXG3 = 0
F = (TXF)	3 = (RX3)	RX3 = TXF – TXD – TXG3: where TXD = 0, TXG3 = 0
G = (TXG)	*Sequential input to receivers {1 + 2 + 3}	RX3 = TXG3 – TXD – TXF: where TXD = 0, TXF = 0

**Algorithm 1.** Relationship between two communication links using *G* = (*V, E*).

Integer E1; /*E one of EI*/
Integer E2; /*E two of EI*/
Integer Communication_Link_Active_Status;
Integer Communication_Active_Link;
Integer Communication_Link_Not_Active_Status = 0;

***Start Program:***
POLLING_TX _ACTIVE_STATUS /*Program location*/
*Poll (Communication_Link_Active_Status);*
*If (Communication_Link_Active_Status == 1);*
*{*
*Goto (ACTIVE_TX_SELECT);*
*}*
*Else {*
*Goto (POLLING_TX _ACTIVE_STATUS);*
*}*
ACTIVE_TX_SELECT /*Program location*/
*While (Communication_Link_Active_Status == 1);*
*{*
*Poll (Communication_Link_Active);*
*If (Communication_Link_Active == 1);*
*{*
*E2 = Communication_Link_Not_Active_Status;*
*}*
*Else*
*{*
*If (Communication_Link_Active == 2);*
*{*
*E1 = Communication_Link_Not_Active_Status;*
*}*
*}*
*Poll (Communication_Link_Active_Status);*
*}*
*Goto (POLLING_TX _ACTIVE_STATUS);*
*End Program;*
*Keys:*
*Goto = Jump to program location (Location Name)*
*Poll = Check the status flag (Status Flag Name)*
